# *N*-{2-[*N*-(4-Methyl­phen­yl)oxamo­yl]phen­yl}propanamide

**DOI:** 10.1107/S1600536810023263

**Published:** 2010-06-23

**Authors:** Humayun Pervez, Maqbool Ahmad, Muhammad Yaqub, M. Nawaz Tahir, Naveeda Saira

**Affiliations:** a Department of Chemistry, Bahauddin Zakariya University, Multan 60800, Pakistan; b Department of Physics, University of Sargodha, Sargodha, Pakistan

## Abstract

The title compound, C_18_H_18_N_2_O_3_, is the product of the heterocyclic ring cleavage at position 2 of 1-propionylisatin. Two centrosymmetric cyclic motifs, *viz. R*_2_^2^(14) and *R*_2_^2^(18), are formed by N—H⋯O hydrogen bonds with the propanamide and amino­phenyl units, respectively, as the N—H donors. These motifs combine into two *C*_2_^2^(8) chain motifs parallel to the *b* axis. The chain structure is stabilized by C—H⋯π inter­actions between the benzene rings, where C—H is from the phenyl ring of the cleaved part of 1-pro­pionylisatin.

## Related literature

For related structures, see: Hohne & Seidel (1979 ▶); Boryczka *et al.* (1998 ▶); Zukerman-Schpector *et al.* (1994 ▶). For synthetic background, see: Pervez *et al.* (2009 ▶, 2010*a* ▶,*b* ▶). For graph-set notation, see: Bernstein *et al.* (1995 ▶).
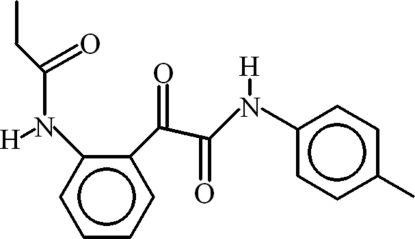


## Experimental

### 

#### Crystal data

C_18_H_18_N_2_O_3_
*M* *_r_* = 310.34
Triclinic, 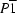

*a* = 9.2048 (4) Å
*b* = 9.7717 (3) Å
*c* = 10.4404 (4) Å
α = 72.962 (2)°
β = 72.920 (1)°
γ = 69.285 (2)°
*V* = 820.63 (6) Å^3^
*Z* = 2
Mo *K*α radiation
μ = 0.09 mm^−1^
*T* = 296 K
0.32 × 0.24 × 0.22 mm

#### Data collection

Bruker Kappa APEXII CCD diffractometer
Absorption correction: multi-scan (*SADABS*; Bruker, 2005 ▶) *T*_min_ = 0.942, *T*_max_ = 0.952
11476 measured reflections
2933 independent reflections
2402 reflections with *I* > 2σ(*I*)
*R*_int_ = 0.023

#### Refinement

*R*[*F* ^2^ > 2σ(*F* ^2^)] = 0.039
*wR*(*F* ^2^) = 0.111
*S* = 1.03
2933 reflections
210 parameters
H-atom parameters constrained
Δρ_max_ = 0.18 e Å^−3^
Δρ_min_ = −0.21 e Å^−3^

### 

Data collection: *APEX2* (Bruker, 2007 ▶); cell refinement: *SAINT* (Bruker, 2007 ▶); data reduction: *SAINT*; program(s) used to solve structure: *SHELXS97* (Sheldrick, 2008 ▶); program(s) used to refine structure: *SHELXL97* (Sheldrick, 2008 ▶); molecular graphics: *ORTEP-3 for Windows* (Farrugia, 1997 ▶) and *PLATON* (Spek, 2009 ▶); software used to prepare material for publication: *WinGX* (Farrugia, 1999 ▶) and *PLATON*.

## Supplementary Material

Crystal structure: contains datablocks global, I. DOI: 10.1107/S1600536810023263/gk2285sup1.cif

Structure factors: contains datablocks I. DOI: 10.1107/S1600536810023263/gk2285Isup2.hkl

Additional supplementary materials:  crystallographic information; 3D view; checkCIF report

## Figures and Tables

**Table 1:** Hydrogen-bond geometry (Å, °) *Cg*1 is the centroid of C1–C6 benzene ring.

*D*—H⋯*A*	*D*—H	H⋯*A*	*D*⋯*A*	*D*—H⋯*A*
N1—H1⋯O2	0.86	2.46	2.7678 (17)	102
N1—H1⋯O3^i^	0.86	2.14	2.9247 (18)	152
N2—H2*A*⋯O1^ii^	0.86	2.07	2.8821 (16)	157
C2—H2⋯O2^i^	0.93	2.58	3.506 (2)	175
C14—H14⋯*Cg*1^ii^	0.93	2.89	3.6693 (18)	142

Symmetry codes: (i) 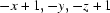; (ii) 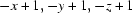.

**Fig. 1.**
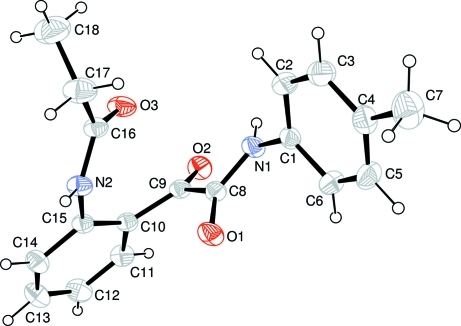
View of the title compound with the atom numbering scheme. The displacement ellipsoids are drawn at the 30% probability level. H-atoms are shown by small circles of arbitrary radii.

**Fig. 2.**
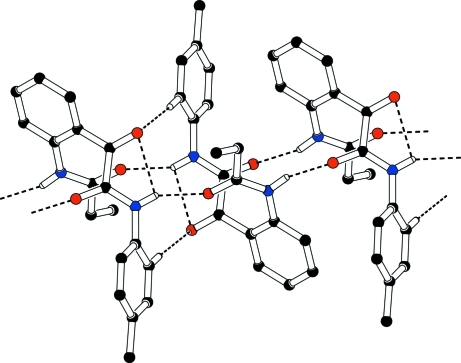
The partial packing (PLATON; Spek, 2009) which shows that molecules arranged via hydrogen bonds into one-dimensional polymeric chains extending along the b axis.

## supplementary crystallographic information

### Comment

We recently have reported the synthesis and crystal structures of certain isatin derivatives (Pervez *et al.*, 2009, 2010*a*, 2010*b*). The title compound (I), (Fig. 1) is the side product obtained in low yield due to the heterocyclic ring cleavage at position-2 of 1-propionylisatin when reacted with *p*-toluidine.

The crystal structures of (II) *i.e.* 2-oxo-*N*,2-diphenylacetamide (Boryczka *et al.*, 1998) and (III) *i.e. p*-tolyl-glyoxylic acid *p*-chloroanilide (Hohne & Seidel, 1979) have been published. The crystal structure of (I) differs from (II) and (III) due to substituants at the phenyl rings. The crystal structure of (IV) *i.e.* 2^'^-(*N*-isopropyloxamoyl)acetanilide (Zukerman-Schpector *et al.*, 1994) has been published, which has isopropyl instead of tolyl and methyl instead of ethyl when compared to (I).

In the crystal structure of (I), the tolylamino group A (C1—C7/N1) and B(C9–C15/N2) of the cleaved part of 1-propionylisatin are planar with r. m. s. deviation of 0.0364 and 0.0456 Å, respectively. The dihedral angle between A/B is 80.25 (5) °. There exist an S(5) ring motif (Bernstein *et al.*, 1995) due to N—H···O interactions (Table 1). In the central part short intramolecular C═O···C═O contact replaces a hydrogen-bond plausible S(6). The central part of (I) has twisting flexibility to set the orientation of substituated phenyl rings. The intermolecular interactions of N—H···O and C—H···O types complete *R*_2_^2^(12) and *R*_2_^2^(18) ring motifs setting the two molecules in dimeric way. These dimers are interlinked through N—H···O interactions with *R*_2_^2^(14) ring motif (Table 1, Fig. 2). The polymeric chain extends along the crystallographic *b* axis. The C—H···π interaction (Table 1) also play role in stabilizing the molecules.

### Experimental

To a refluxing solution of 1-propionylisatin (1.02 g, 5 mmol) in ethanol (15 ml) containing 2–3 drops of concentrated sulfuric acid was added the solution of *p*-toluidine (0.54 g, 5 mmol) made in ethanol (5 ml). The reaction mixture was then refluxed for 2 h, after which it was left at room temperature overnight. The reddish yellow solid formed was collected by suction filtration, washing of which with ethanol to get rid of the soluble impurities, however, gave a dirty white solid. Recrystallization of the same from ethanol furnished the title heterocyclic ring cleavage product (I) in pure form (0.33 g, 21%) m.p. 423 K. The single crystals of (I) for *x*-ray analysis were grown in ethyl acetate-petroleum ether (1:4) by diffusion method at room temperature.

### Refinement

The H-atoms were positioned geometrically (N–H = 0.86 Å, C–H = 0.93–0.97 Å) and refined as riding with *U*_iso_(H) = *xU*_eq_(C, N), where *x* = 1.5 for methyl and *x* = 1.2 for all other H-atoms. !5

### Crystal data

**Table:**

C_18_H_18_N_2_O_3_	*Z* = 2
*M**_r_* = 310.34	*F*(000) = 328
Triclinic, *P*1	*D*_x_ = 1.254 Mg m^−^^3^
Hall symbol: -P 1	Mo *K*α radiation, λ = 0.71073 Å
*a* = 9.2048 (4) Å	Cell parameters from 2402 reflections
*b* = 9.7717 (3) Å	θ = 2.8–25.3°
*c* = 10.4404 (4) Å	µ = 0.09 mm^−^^1^
α = 72.962 (2)°	*T* = 296 K
β = 72.920 (1)°	Prism, yellow
γ = 69.285 (2)°	0.32 × 0.24 × 0.22 mm
*V* = 820.63 (6) Å^3^	

### Data collection

**Table:**

Bruker Kappa APEXII CCD diffractometer	2933 independent reflections
Radiation source: fine-focus sealed tube	2402 reflections with *I* > 2σ(*I*)
graphite	*R*_int_ = 0.023
Detector resolution: 8.2 pixels mm^-1^	θ_max_ = 25.3°, θ_min_ = 2.8°
ω scans	*h* = −11→10
Absorption correction: multi-scan (*SADABS*; Bruker, 2005)	*k* = −11→11
*T*_min_ = 0.942, *T*_max_ = 0.952	*l* = −12→12
11476 measured reflections	

### Refinement

**Table:**

Refinement on *F*^2^	Primary atom site location: structure-invariant direct methods
Least-squares matrix: full	Secondary atom site location: difference Fourier map
*R*[*F*^2^ > 2σ(*F*^2^)] = 0.039	Hydrogen site location: inferred from neighbouring sites
*wR*(*F*^2^) = 0.111	H-atom parameters constrained
*S* = 1.03	*w* = 1/[σ^2^(*F*_o_^2^) + (0.0538*P*)^2^ + 0.1939*P*] where *P* = (*F*_o_^2^ + 2*F*_c_^2^)/3
2933 reflections	(Δ/σ)_max_ < 0.001
210 parameters	Δρ_max_ = 0.18 e Å^−^^3^
0 restraints	Δρ_min_ = −0.21 e Å^−^^3^

### Special details

**Table:**

Geometry. Bond distances, angles *etc*. have been calculated using the rounded fractional coordinates. All su's are estimated from the variances of the (full) variance-covariance matrix. The cell e.s.d.'s are taken into account in the estimation of distances, angles and torsion angles
Refinement. Refinement of *F*^2^ against ALL reflections. The weighted *R*-factor *wR* and goodness of fit *S* are based on *F*^2^, conventional *R*-factors *R* are based on *F*, with *F* set to zero for negative *F*^2^. The threshold expression of *F*^2^ > σ(*F*^2^) is used only for calculating *R*-factors(gt) *etc*. and is not relevant to the choice of reflections for refinement. *R*-factors based on *F*^2^ are statistically about twice as large as those based on *F*, and *R*- factors based on ALL data will be even larger.

### Fractional atomic coordinates and isotropic or equivalent isotropic displacement parameters (Å^2^)

**Table:**

	*x*	*y*	*z*	*U*_iso_*/*U*_eq_	
O1	0.73296 (14)	0.26832 (11)	0.51591 (11)	0.0532 (4)	
O2	0.60422 (14)	−0.02427 (11)	0.72653 (12)	0.0527 (4)	
O3	0.36026 (15)	0.18977 (12)	0.55159 (12)	0.0572 (4)	
N1	0.73440 (15)	0.06472 (13)	0.45212 (13)	0.0454 (4)	
N2	0.36473 (15)	0.41064 (13)	0.57412 (12)	0.0434 (4)	
C1	0.81861 (17)	0.09987 (16)	0.31438 (16)	0.0423 (5)	
C2	0.7508 (2)	0.11442 (19)	0.20806 (17)	0.0533 (6)	
C3	0.8280 (2)	0.1562 (2)	0.07434 (18)	0.0584 (6)	
C4	0.9720 (2)	0.18504 (18)	0.04359 (18)	0.0541 (6)	
C5	1.0394 (2)	0.1665 (2)	0.15214 (19)	0.0585 (6)	
C6	0.96495 (19)	0.12374 (19)	0.28597 (18)	0.0533 (6)	
C7	1.0503 (3)	0.2377 (3)	−0.1028 (2)	0.0789 (8)	
C8	0.69138 (17)	0.15593 (15)	0.53889 (15)	0.0396 (5)	
C9	0.58882 (17)	0.10771 (15)	0.67838 (15)	0.0404 (5)	
C10	0.49283 (17)	0.22541 (16)	0.75877 (14)	0.0394 (4)	
C11	0.5065 (2)	0.19194 (19)	0.89436 (16)	0.0503 (5)	
C12	0.4344 (2)	0.2961 (2)	0.97574 (17)	0.0606 (6)	
C13	0.3468 (2)	0.4365 (2)	0.92194 (18)	0.0619 (6)	
C14	0.3263 (2)	0.47161 (18)	0.78955 (17)	0.0513 (5)	
C15	0.39731 (17)	0.36707 (15)	0.70660 (14)	0.0390 (4)	
C16	0.33857 (18)	0.32494 (17)	0.50734 (16)	0.0444 (5)	
C17	0.2851 (3)	0.4048 (2)	0.3751 (2)	0.0701 (7)	
C18	0.1504 (3)	0.3664 (3)	0.3605 (3)	0.0991 (10)	
H1	0.71043	−0.01814	0.48038	0.0544*	
H2	0.65336	0.09623	0.22615	0.0639*	
H2A	0.36115	0.50119	0.53138	0.0521*	
H3	0.78158	0.16501	0.00316	0.0701*	
H5	1.13748	0.18326	0.13421	0.0702*	
H6	1.01320	0.11098	0.35712	0.0640*	
H7A	1.16236	0.18772	−0.11688	0.1184*	
H7B	1.03271	0.34386	−0.12098	0.1184*	
H7C	1.00549	0.21520	−0.16359	0.1184*	
H11	0.56593	0.09679	0.93107	0.0604*	
H12	0.44498	0.27149	1.06627	0.0727*	
H13	0.30103	0.50856	0.97527	0.0744*	
H14	0.26406	0.56636	0.75514	0.0616*	
H17A	0.25468	0.51191	0.36884	0.0841*	
H17B	0.37380	0.38080	0.29937	0.0841*	
H18A	0.06159	0.39040	0.43466	0.1487*	
H18B	0.18084	0.26134	0.36232	0.1487*	
H18C	0.12098	0.42260	0.27493	0.1487*	

### Atomic displacement parameters (Å^2^)

**Table:**

	*U*^11^	*U*^22^	*U*^33^	*U*^12^	*U*^13^	*U*^23^
O1	0.0656 (7)	0.0375 (6)	0.0605 (7)	−0.0269 (5)	−0.0023 (5)	−0.0126 (5)
O2	0.0635 (7)	0.0321 (5)	0.0596 (7)	−0.0167 (5)	−0.0155 (5)	0.0002 (5)
O3	0.0757 (8)	0.0411 (6)	0.0698 (8)	−0.0238 (5)	−0.0319 (6)	−0.0087 (5)
N1	0.0516 (8)	0.0360 (6)	0.0531 (8)	−0.0213 (6)	−0.0045 (6)	−0.0122 (5)
N2	0.0566 (8)	0.0321 (6)	0.0465 (7)	−0.0200 (5)	−0.0182 (6)	0.0002 (5)
C1	0.0417 (8)	0.0338 (7)	0.0522 (9)	−0.0120 (6)	−0.0048 (7)	−0.0143 (6)
C2	0.0465 (9)	0.0610 (10)	0.0617 (11)	−0.0255 (8)	−0.0069 (8)	−0.0191 (8)
C3	0.0581 (11)	0.0693 (11)	0.0555 (10)	−0.0246 (9)	−0.0114 (8)	−0.0176 (8)
C4	0.0508 (10)	0.0466 (9)	0.0596 (10)	−0.0144 (7)	−0.0001 (8)	−0.0152 (7)
C5	0.0408 (9)	0.0644 (11)	0.0714 (12)	−0.0227 (8)	−0.0031 (8)	−0.0159 (9)
C6	0.0442 (9)	0.0586 (10)	0.0622 (11)	−0.0185 (8)	−0.0124 (8)	−0.0147 (8)
C7	0.0777 (14)	0.0777 (14)	0.0698 (13)	−0.0306 (11)	0.0063 (11)	−0.0111 (10)
C8	0.0420 (8)	0.0293 (7)	0.0491 (9)	−0.0116 (6)	−0.0126 (7)	−0.0061 (6)
C9	0.0439 (8)	0.0325 (7)	0.0494 (9)	−0.0153 (6)	−0.0170 (7)	−0.0032 (6)
C10	0.0423 (8)	0.0368 (7)	0.0419 (8)	−0.0184 (6)	−0.0089 (6)	−0.0040 (6)
C11	0.0582 (10)	0.0475 (9)	0.0462 (9)	−0.0181 (7)	−0.0179 (8)	−0.0013 (7)
C12	0.0765 (12)	0.0680 (12)	0.0415 (9)	−0.0242 (10)	−0.0147 (8)	−0.0118 (8)
C13	0.0784 (13)	0.0568 (10)	0.0527 (10)	−0.0194 (9)	−0.0076 (9)	−0.0215 (8)
C14	0.0591 (10)	0.0386 (8)	0.0556 (10)	−0.0141 (7)	−0.0101 (8)	−0.0115 (7)
C15	0.0436 (8)	0.0345 (7)	0.0428 (8)	−0.0189 (6)	−0.0098 (6)	−0.0039 (6)
C16	0.0481 (9)	0.0417 (8)	0.0497 (9)	−0.0199 (7)	−0.0148 (7)	−0.0063 (7)
C17	0.0917 (14)	0.0686 (12)	0.0623 (12)	−0.0303 (11)	−0.0355 (11)	−0.0043 (9)
C18	0.1088 (19)	0.0778 (15)	0.133 (2)	−0.0164 (13)	−0.0782 (18)	−0.0158 (14)

### Geometric parameters (Å, °)

**Table:**

O1—C8	1.2244 (19)	C12—C13	1.371 (3)
O2—C9	1.2119 (18)	C13—C14	1.376 (2)
O3—C16	1.228 (2)	C14—C15	1.391 (2)
N1—C1	1.425 (2)	C16—C17	1.501 (3)
N1—C8	1.3359 (19)	C17—C18	1.475 (4)
N2—C15	1.4116 (19)	C2—H2	0.9300
N2—C16	1.352 (2)	C3—H3	0.9300
N1—H1	0.8600	C5—H5	0.9300
N2—H2A	0.8600	C6—H6	0.9300
C1—C6	1.380 (3)	C7—H7A	0.9600
C1—C2	1.376 (2)	C7—H7B	0.9600
C2—C3	1.382 (2)	C7—H7C	0.9600
C3—C4	1.379 (3)	C11—H11	0.9300
C4—C7	1.508 (3)	C12—H12	0.9300
C4—C5	1.384 (3)	C13—H13	0.9300
C5—C6	1.376 (3)	C14—H14	0.9300
C8—C9	1.529 (2)	C17—H17A	0.9700
C9—C10	1.490 (2)	C17—H17B	0.9700
C10—C11	1.390 (2)	C18—H18A	0.9600
C10—C15	1.402 (2)	C18—H18B	0.9600
C11—C12	1.377 (2)	C18—H18C	0.9600
			
O1···N2	3.1213 (19)	C3···H13^i^	3.0000
O1···C6	3.027 (2)	C6···H14^i^	3.0100
O1···C15	3.138 (2)	C8···H6	2.9700
O1···N2^i^	2.8821 (16)	C10···H7A^vi^	3.0400
O2···N1	2.7678 (17)	C11···H11^iii^	3.0500
O2···O3	3.0986 (18)	C11···H3^vii^	2.9700
O3···C8	2.914 (2)	C12···H3^vii^	3.0600
O3···N1^ii^	2.9247 (18)	C15···H7A^vi^	3.1000
O3···C10	2.930 (2)	H1···O2	2.4600
O3···C9	2.577 (2)	H1···O3^ii^	2.1400
O3···N1	3.178 (2)	H1···H18B^ii^	2.5200
O3···O2	3.0986 (18)	H2···O2^ii^	2.5800
O1···H6	2.8200	H2A···H14	2.4300
O1···H14^i^	2.8200	H2A···H17A	2.1600
O1···H17A^i^	2.8000	H2A···O1^i^	2.0700
O1···H2A^i^	2.0700	H2A···N2^i^	2.7700
O2···H12^iii^	2.8000	H2A···H2A^i^	2.4400
O2···H1	2.4600	H3···C11^viii^	2.9700
O2···H11	2.6100	H3···C12^viii^	3.0600
O2···H2^ii^	2.5800	H3···H7C	2.3700
O2···H5^iv^	2.8800	H5···H7A	2.5500
O2···H18B^ii^	2.6600	H5···O2^iv^	2.8800
O3···H18B	2.7200	H6···O1	2.8200
O3···H1^ii^	2.1400	H6···C8	2.9700
N1···O2	2.7678 (17)	H6···H18B^ix^	2.5000
N1···O3	3.178 (2)	H7A···C10^x^	3.0400
N1···O3^ii^	2.9247 (18)	H7A···C15^x^	3.1000
N2···O1	3.1213 (19)	H7A···H5	2.5500
N2···C8	3.157 (2)	H7B···H18C^xi^	2.5900
N2···O1^i^	2.8821 (16)	H7C···H3	2.3700
N2···N2^i^	3.298 (2)	H11···O2	2.6100
N2···H2A^i^	2.7700	H11···C11^iii^	3.0500
C3···C4^v^	3.572 (3)	H11···H11^iii^	2.4700
C3···C7^v^	3.554 (3)	H12···O2^iii^	2.8000
C4···C3^v^	3.572 (3)	H13···C3^i^	3.0000
C6···O1	3.027 (2)	H14···H2A	2.4300
C7···C3^v^	3.554 (3)	H14···O1^i^	2.8200
C8···C16	3.146 (2)	H14···C1^i^	2.9900
C8···N2	3.157 (2)	H14···C6^i^	3.0100
C8···O3	2.914 (2)	H17A···H2A	2.1600
C9···C16	3.120 (2)	H17A···O1^i^	2.8000
C9···O3	2.577 (2)	H18B···O3	2.7200
C10···O3	2.930 (2)	H18B···H6^xii^	2.5000
C15···O1	3.138 (2)	H18B···O2^ii^	2.6600
C16···C8	3.146 (2)	H18B···H1^ii^	2.5200
C16···C9	3.120 (2)	H18C···H7B^xi^	2.5900
C1···H14^i^	2.9900		
			
C1—N1—C8	122.33 (13)	C16—C17—C18	113.78 (19)
C15—N2—C16	126.83 (13)	C1—C2—H2	120.00
C8—N1—H1	119.00	C3—C2—H2	120.00
C1—N1—H1	119.00	C2—C3—H3	119.00
C16—N2—H2A	117.00	C4—C3—H3	119.00
C15—N2—H2A	117.00	C4—C5—H5	119.00
C2—C1—C6	119.50 (16)	C6—C5—H5	119.00
N1—C1—C6	121.04 (15)	C1—C6—H6	120.00
N1—C1—C2	119.43 (16)	C5—C6—H6	120.00
C1—C2—C3	119.74 (18)	C4—C7—H7A	109.00
C2—C3—C4	121.78 (18)	C4—C7—H7B	109.00
C3—C4—C7	120.86 (19)	C4—C7—H7C	109.00
C5—C4—C7	121.7 (2)	H7A—C7—H7B	109.00
C3—C4—C5	117.39 (17)	H7A—C7—H7C	110.00
C4—C5—C6	121.66 (19)	H7B—C7—H7C	109.00
C1—C6—C5	119.89 (17)	C10—C11—H11	119.00
O1—C8—N1	124.88 (14)	C12—C11—H11	119.00
N1—C8—C9	115.40 (13)	C11—C12—H12	120.00
O1—C8—C9	119.66 (13)	C13—C12—H12	120.00
O2—C9—C8	119.57 (13)	C12—C13—H13	120.00
O2—C9—C10	122.47 (14)	C14—C13—H13	120.00
C8—C9—C10	117.22 (12)	C13—C14—H14	120.00
C11—C10—C15	118.54 (14)	C15—C14—H14	120.00
C9—C10—C11	116.22 (14)	C16—C17—H17A	109.00
C9—C10—C15	125.18 (13)	C16—C17—H17B	109.00
C10—C11—C12	121.67 (16)	C18—C17—H17A	109.00
C11—C12—C13	119.31 (16)	C18—C17—H17B	109.00
C12—C13—C14	120.45 (17)	H17A—C17—H17B	108.00
C13—C14—C15	120.80 (16)	C17—C18—H18A	109.00
N2—C15—C10	123.90 (13)	C17—C18—H18B	109.00
N2—C15—C14	116.97 (13)	C17—C18—H18C	109.00
C10—C15—C14	119.12 (14)	H18A—C18—H18B	109.00
O3—C16—C17	122.47 (16)	H18A—C18—H18C	109.00
N2—C16—C17	115.84 (14)	H18B—C18—H18C	109.00
O3—C16—N2	121.68 (15)		
			
C8—N1—C1—C2	−118.93 (18)	N1—C8—C9—C10	−159.46 (15)
C8—N1—C1—C6	59.2 (2)	O1—C8—C9—O2	−147.04 (17)
C1—N1—C8—O1	−8.3 (3)	C8—C9—C10—C15	47.4 (2)
C1—N1—C8—C9	174.65 (14)	O2—C9—C10—C11	40.4 (2)
C15—N2—C16—O3	−9.2 (3)	O2—C9—C10—C15	−142.61 (18)
C16—N2—C15—C10	38.2 (3)	C8—C9—C10—C11	−129.66 (17)
C16—N2—C15—C14	−140.31 (18)	C15—C10—C11—C12	−2.8 (3)
C15—N2—C16—C17	172.10 (17)	C9—C10—C15—N2	7.8 (3)
C2—C1—C6—C5	2.1 (3)	C9—C10—C15—C14	−173.71 (16)
N1—C1—C2—C3	176.60 (15)	C9—C10—C11—C12	174.46 (17)
C6—C1—C2—C3	−1.6 (3)	C11—C10—C15—N2	−175.24 (16)
N1—C1—C6—C5	−176.03 (15)	C11—C10—C15—C14	3.3 (3)
C1—C2—C3—C4	−0.4 (3)	C10—C11—C12—C13	0.0 (3)
C2—C3—C4—C7	−177.02 (19)	C11—C12—C13—C14	2.3 (3)
C2—C3—C4—C5	1.8 (3)	C12—C13—C14—C15	−1.8 (3)
C3—C4—C5—C6	−1.2 (3)	C13—C14—C15—N2	177.53 (17)
C7—C4—C5—C6	177.57 (19)	C13—C14—C15—C10	−1.1 (3)
C4—C5—C6—C1	−0.7 (3)	O3—C16—C17—C18	47.0 (3)
O1—C8—C9—C10	23.3 (2)	N2—C16—C17—C18	−134.27 (19)
N1—C8—C9—O2	30.2 (2)		

Symmetry codes: (i) −*x*+1, −*y*+1, −*z*+1; (ii) −*x*+1, −*y*, −*z*+1; (iii) −*x*+1, −*y*, −*z*+2; (iv) −*x*+2, −*y*, −*z*+1; (v) −*x*+2, −*y*, −*z*; (vi) *x*−1, *y*, *z*+1; (vii) *x*, *y*, *z*+1; (viii) *x*, *y*, *z*−1; (ix) *x*+1, *y*, *z*; (x) *x*+1, *y*, *z*−1; (xi) −*x*+1, −*y*+1, −*z*; (xii) *x*−1, *y*, *z*.

### Hydrogen-bond geometry (Å, °)

**Table:**

Cg1 is the centroid of C1–C6 benzene ring.

**Table:**

*D*—H···*A*	*D*—H	H···*A*	*D*···*A*	*D*—H···*A*
N1—H1···O2	0.86	2.46	2.7678 (17)	102
N1—H1···O3^ii^	0.86	2.14	2.9247 (18)	152
N2—H2A···O1^i^	0.86	2.07	2.8821 (16)	157
C2—H2···O2^ii^	0.93	2.58	3.506 (2)	175
C14—H14···Cg1^i^	0.93	2.89	3.6693 (18)	142

Symmetry codes: (ii) −*x*+1, −*y*, −*z*+1; (i) −*x*+1, −*y*+1, −*z*+1.
